# 624. Probability of Target Attainment of Ceftolozane/Tazobactam Among Adult Patients with Hospital-Acquired Pneumonia/Ventilator-Associated Pneumonia Secondary to *Pseudomonas aeruginosa* in Latin America

**DOI:** 10.1093/ofid/ofac492.676

**Published:** 2022-12-15

**Authors:** Aline Okuma, Thales Polis, Jacqueline Pavia, Gustavo Mizuno, Jacqueline Ferrari, Charles A DeRyke

**Affiliations:** MSD Brazil, Sao Paulo, Brazil, Sao Paulo, Sao Paulo, Brazil; MSD Brazil, Sao Paulo, Brazil, Sao Paulo, Sao Paulo, Brazil; MSD Colombia, Bogota, Colombia., Bogota, Distrito Capital de Bogota, Colombia; MSD Brazil, Sao Paulo, Brazil, Sao Paulo, Sao Paulo, Brazil; MSD Brazil, Sao Paulo, Brazil, Sao Paulo, Sao Paulo, Brazil; Merck Research Laboratories, Longwood, Florida

## Abstract

**Background:**

*Pseudomonas aeruginosa (Pa)* is a common cause of nosocomial pneumonia; resistance among traditionally used empiric agents is observed frequently. To improve patient outcomes, a heightened focus has been placed on evaluating the adequacy of recommended dosing regimens, particularly of beta-lactams. The Phase 3 study ASPECT-NP demonstrated the efficacy and safety of 3 g of ceftolozane/tazobactam (C/T) infused every 8 h for 8 to 14 days for treatment of adults with Hospital-Acquired Pneumonia/Ventilator-Associated Pneumonia (HAP/VAP). We assessed the Probability of Target Attainment (PTA) of the C/T 3g dosage regimen in patients with HAP/VAP due to *Pa*, with an additional focus on carbapenem-resistant *Pa,* from Latin America.

**Methods:**

Non duplicate *Pa* isolates from a respiratory source were collected as part of the SMART surveillance program from 36 sites in 10 Latin American countries during 2017-2020. MICs were determined by broth microdilution and interpreted by CLSI criteria. C and T concentration-time profiles were simulated in plasma and ELF following administration of the approved 3g (2g/1g) C/T dose (or equivalent dose adjusted based on renal function) administered by 1-hour infusion every 8 hours. PTA in plasma and ELF was calculated using the PK/PD target of 30% *f*T > MIC for C. T does not contribute to the antipseudomonal activity of C and therefore was not considered.

**Results:**

A total of 2,757 *P. aeruginosa* were collected, of which 1208 (43.8%) were carbapenem nonsusceptible. C/T susceptiblity was 87.7% against all *Pa* and 73.4% among carbapenem nonsusceptible strains. At C/T doses of 2g/1g (CrCL > 50mL/min); 1g/0.5g (30mL/min≤ CrCL≤ 50mL/min), and 500mg/250mg (15mL/min≤ CrCL≤29mL/min), steady-state ceftolozane plasma and ELF PTA was 100% and > 99%, respectively, for isolates with an MIC at the *Pa* susceptiblity breakpoint of 4 µg/mL. Ceftolozane plasma and ELF PTA remained above 90% up to an MIC of 16 µg/mL and 8 µg/mL, respectively.

**Conclusion:**

The regulatory approved pneumonia dosage regimen of C/T 3g (administered over 1 hour) every 8 hours (or equivalent dose adjusted based on renal function) resulted in high plasma and ELF PTAs sufficient to cover the vast majority of circulating *P. aeruginosa* present in Latin America, including carbapenem resistant strains.

**Disclosures:**

**Aline Okuma, PharmD**, MSD Brazil: Employee **Thales Polis, MD**, MSD Brazil: Employee **Jacqueline Pavia, MD**, MSD COLOMBIA: EMPLOYEE **Gustavo Mizuno, PharmD**, MSD Brazil: Employee **Jacqueline Ferrari, MD**, MSD Brazil: Employee **Charles A. DeRyke, PharmD**, Merck & Co., Inc. Merck Research Laboratories: Stocks/Bonds.

